# The Egyptian Dementia Network (EDN) registry: Towards discovering novel biomarkers of early dementia

**DOI:** 10.1002/alz70856_100261

**Published:** 2025-12-25

**Authors:** Mohamed Salama

**Affiliations:** ^1^ The American University in Cairo, Cairo, Egypt

## Abstract

Dementia and aging problems are currently growing worldwide and are considered public health priorities. The availability of epidemiological and clinical data on the disease greatly impacts our understanding of the disease and, hence, improves disease management and prevention strategies. As part of the development of a national dementia registry for Egypt that would be of great importance as we currently lack the exact epidemiological data and rely only on estimates, the present study is aiming to pilot a cohort for the first national registry for dementia in Egypt, The Egyptian Dementia Network (EDN) registry. EDN will provide a huge set of data as a promising platform for analyzing the prevalence, incidence, and major causes of the disease in Egypt. We aim to coordinate the track of early‐onset AD (EOAD) and vascular dementia cases, which have recently shown a rising pattern. Aiming at 100 cases from each cohort and a similar number of matched controls, we will then develop a tool to register their data. As a research registry, the main aim is to start understanding the causes, mechanisms, and courses of dementia in Egypt. One powerful tool in this regard is peripheral biomarkers. Due to limited access to brain samples, accurate diagnosis of neurodegenerative diseases is still a challenging process. The recent advances in biomarker development are helping to improve diagnostic accuracy through blood samples. The Collected patients’ clinical samples will be used to develop a pilot for proteomic, metabolomics and genomic profiling to measure the peripheral biomarkers and discover the biomarkers related to the Egyptian population. Examining the biomarkers in the Egyptian cohort will serve different goals; first improving diagnostic accuracy, enhancing the ability to differentiate between different phenotypes, and final –long‐term goal‐ to validate the predictive ability of these biomarkers on the Egyptian HCAP (Harmonized Cognitive Assessment Protocol), where the ability of biomarkers to predict future dementia patients will be tested on a longitudinal cohort of aging population.

